# A five-year retrospective review of the maternal and feotal outcome of obstructed labour and its determinants in a tertiary hospital in Nigeria

**DOI:** 10.4314/ahs.v22i2.58

**Published:** 2022-06

**Authors:** Chidebe C Anikwe, Cyril C Ikeoha, Christian O Ogah, Collins A Kalu, Ifeyinwa H Anikwe

**Affiliations:** 1 Department of Obstetrics and Gynaecology, Federal Teaching Hospital Abakaliki Ebonyi state; 2 Department of Obstetrics and Gynaecology, Kubwa General Hospital Abuja Nigeria; 3 Department of Administration, Federal Teaching Hospital Abakaliki Ebonyi state

**Keywords:** Obstructed labour, cephalopelvic disproportion, antenatal care, sepsis, haemorrhage, uterine rupture, Abakaliki

## Abstract

**Background:**

Obstructed labour is one of the common obstetric emergencies in Nigeria which is associated with an increased maternal and foetal complications.

**Objective:**

To determine the maternal and foetal outcome of obstructed labour and its determinants in a tertiary hospital in Ebonyi State University Teaching hospital Abakaliki

**Methods:**

A retrospective review of all women with obstructed labour managed at Ebonyi State University Teaching hospital Abakaliki between January 2007 and December 2011 was carried out.

**Results:**

The prevalence rate of obstructed labour was 3.4% (95%CI 3.37 – 3.42) for the period under review. Women in their second and third decade of life formed 91.6% (196/214) of patients in the study. Majority of obstructed labour occurred in primiparous women (92/214, 42.9%) and the commonest cause of obstructed labour was cephalopelvic disproportion (106/214, 49.6%). The commonest maternal complication was wound infection accounting for 23.2% (48/214) of all the complications. Most of the babies delivered had a good Apgar score as was noted in 60.3% (129/214) of cases. Being unbooked, para 3 and above, maternal age of 30 and above, having no formal education and rural residence were strongly associated with parturient having maternal complication (P > 0.05) and abnormal APGAR score. The maternal and perinatal mortality rate was 191/100,000 live births and 168/1000 deliveries respectively.

**Conclusion:**

The commonest cause of obstructed labour in our review is cephalopelvic disproportion occurring more in primiparous women. Wound infection is the commonest maternal complication with majority of the neonates having a good outcome. Provision of free antenatal care services, education of women on the importance of antenatal care, early presentation in the hospital and early use of broad spectrum antibiotics would help to reduce the associated complications.

## Introduction

Obstructed labour is a common obstetric problem in developing countries 1. It is said to occur when a parturient fails to achieve vaginal delivery despite adequate uterine contraction as a result of mechanical factor necessitating surgical intervention to be taken to prevent injury to the mother and/or foetus. It is one of the preventable causes of maternal and perinatal mortality and morbidity in developing countries. The rate of maternal death in Nigeria is unacceptably high. It was reported to be 814 per 100,000 live births 2 but the rate varies widely. An incidence rate of 430 per 100,000 live births^3^, 840/100,000 live birth^4^, 902.7/100,000 live births^5^, 1513.4 per 100,000 live births^6^, 636/100,000 deliveries^7^, 740/100,000 deliveries^8^ and 2849/100,000 deliveries^9^ have been reported in Nigeria. Some of the causes of maternal death in our environment include postpartum haemorrhage (PPH), preeclampsia/eclampsia, the complication of unsafe abortion, sepsis and obstructed labour^10^. The number of maternal deaths as a result of obstructed labour and/or rupture of the uterus varies between 4% and 70%, amounting to a maternal mortality rate as high as 410/100,000 live births^11^.The risk of maternal death from obstructed labour is greatest in developing countries with poorly resourced health services^12^. Developed worlds were able to overcome this problem as a result of improvement in the standard of living, women empowerment and efficient maternal and child health care delivery system^13^. This is anchored on proper utilization of antenatal care and labour services which is managed by a skilled birth attendance 13. Belief in-home deliveries supervised by un-trained health personnel, aversion to surgery and uneven distribution of the minimal health care delivery facility are some of the contibuting factors in our environment^14^.

Cephalopelvic disproportion due to the contracted pelvis is the commonest cause of the obstructed labour in Nigeria^14^. Other causes include mal-positioning, foetal macrosomia, hydrocephalus, impacted transverse lie, malpresentation, shoulder dystocia and locked twins^15^. Pelvic contraction results from inadequate growth due to malnutrition and ill health, prevalent in childhood among poor nation^12^. Adequate prevention, however, can be achieved only through a multidisciplinary approach aimed in the short term at identifying high-risk cases and in the long term at improving nutrition^16^. Even though there has been an improvement in the percentage of labour coverage by skilled birth attendant globally, the percentage coverage in sub-Saharan African, with the greatest burden of bad obstetric outcome, is low compared to developed world 13. In Chad, Niger, Nigeria, Mali, Cameroon and Ghana for example, skilled birth attendant coverage was estimated to be 24.3%, 39.1%, 43.3%, 67.3%, 69%, and 78.9% respectively unlike in developed world that had more than 90% coverage^13^. This finding is one of the contributing factors to the increased burden of obstructed labour and its complications in our environment. It has been estimated that to reduce the global maternal mortality ratio to less than 70 per 100,000 live births as envisaged in Sustainable Development Goals (SDGs) that antenatal coverage should increase, 81% of delivery should be in a functional health facility with 87% of skilled birth attendance^17^. This is still a distant dream in Nigeria which we hope to achieve. On this background, this study is aimed at evaluating the maternal and foetal outcome of the women with obstructed labour managed in our facility. It will assist also to determine the factors influencing these outcomes.

## Materials and Methods

### Study design and setting

This retrospective study was carried out in the obstetric department of Ebonyi state University Teaching Hospital, Abakaliki, Ebonyi State between 1st January 2007 and 31st December 2011. Ebonyi State is one of the states in the southeast of Nigeria. It has a total land mass of 5,533km^2^ and a population of about 2, 176, 947 million. It has 13 local governments, one urban, one semi-urban and other rural. The major occupation of the population is farming. Ebonyi state teaching Hospital, Abakaliki is the only teaching hospital in the state receiving a referral from a private hospital, a mission hospital in the state and from neighbouring states. The Department of Obstetrics and Gynaecology is managed by 11 trained consultants and Resident doctors with the help of trained nurses.

### Study population

All deliveries conducted in the Department of Obstetrics and Gynaecology of Ebonyi state University Teaching Hospital between 1^st^ January 2007 and 31^st^ December 2011 were included in the study. The total number of women delivered was 6294, of these, 214 had obstructed labour and were studied. All mothers who were admitted to the labour ward with a diagnosis of obstructed labour during the study period were included in the study. The labour is managed by a team of resident doctors led by a senior registrar under the supervision of a labour ward consultant. The obstetric emergency unit is similarly manned as in labour ward. They are assisted by trained midwives. Parturients in labour were admitted in the labour ward when the cervical dilatation is 4 centimeter and above while those for induction of labour were admitted when the Bishop score is 6 and above. They were managed with individualized partograph according to the Departmental protocol. A diagnosis of obstructed labour was made in a parturient with poor progress of labour evidenced by lack of foetal descent, the arrest of cervical dilatation - cervicogram crossing or not crossing the action line, increasing caput and mouldig despite adequacy of uterine contraction 18, 19. For the unbooked parturient who were referred to the facility for failure to deliver having been in labour in a maternity centre outside the facility were admitted and assessed in the obstetric emergency unit of the hospital. A diagnosis of obstructed labour was made in an unbooked parturient with history of failure to deliver in the referral centre after labour augmentation with oxytocin, the presence of three-tumour-abdomen caused by Bandl's ring on the uterus with a full bladder, abnormal lie and mal-presentation^20^. This is supported by a vaginal examination finding of an abnormal pelvis, and/or excessive caput succedaneum and moulding. The folders of these women with obstructed labour were retrieved from the record department after their hospital numbers had been gotten from the general obstetric record book of the department. Data were gathered by Residents in the Department of Obstetrics and Gynaecology from patients' records. Data was collected using a questionnaire and checklist. Information obtained included socio-demographic characteristics of the women, the parity, booking status, the cause of labour obstruction, the mode of delivery, blood loss, foetal weight, Apgar score, maternal/neonatal complication and hospital stay. Ethical approval was obtained for the study from the Research and Ethics committee of the hospital (EBSUTH/REC/VOL1/2011).

### Data analysis

The data was collected in a spreadsheet of our personal computer and analyzed using IBM SPSS Statistics version 20 (IBM Corp., Armonk, NY, USA). Categorical and continuous variables were described with Frequency tables, Percentages, Range and Bar/Pie chart whichever applies. Logistic regression was used to examine the association between socio-demographic /obstetric characteristics of the study population and maternal and foetal complications. Data obtained were coded as follows: Booking – booked = 0, unbooked = 1; Parity – 0–2 = 0, 3–5 =1; Age - < 30 years = 0, ≥ 30 years = 1; Formal education – yes =0, no =1; Marital status - married = 0, unmarried =1; Residence – urban = 0; rural =1; Puerperal sepsis – present = yes, absent = no; Uterine rupture – present = yes, absent = no, PPH - present = yes, absent = no and APGAR score – normal ≥7, abnormal <7. The odds ratios with their 95% CI were calculated to determine the strength and presence of an association. A p-value of ≤ 0.05 was considered significant. Odds ratio (OR) < 1 infers that persons in that category have a lower Odds of maternal and foetal complications while OR > 1 is designated increased probability of maternal and foetal complications.

## Results

Six thousand, two hundred and ninety-four (6,294) women in labour were managed and delivered during the period of study (Jan. 2007 – Dec. 2011). Two hundred and fourteen (214) of these were obstructed labour patients giving a point prevalence rate of 3.4% (95%CI 3.37 – 3.42). During the same period, there were 486 caesarean sections. Obstructed labour accounted for 32.3% (157/486) of these caesarean sections. The patients were mostly in lower socio-economic class as 136 (63.6%) were illiterate or attended primary school with only 24(11.2%) of their husband being professional, petty trader or politicians.

Table 1 shows the sociodemographic and obstetrics characteristic of the study population. The age range was between 15 and 43 years, fourteen (6.6%) were teenagers while 4 (1.9%) were above 40 years. One hundred and fifty-six (72.9%) of the patient were unbooked and received no antenatal care in any health care facilities. The main reason adduced for lack of antenatal care was the inability to pay for hospital care; One hundred and two (47.7%) were referred from another maternity centre while 58 (27.1%) had antenatal care at our facility.

**Table 1:** Socio-demographic and obstetrics characteristic of the study population

Characteristics	Number	Frequency
**Age**		
15–19	14	6.6
20–24	32	14.9
25–29	112	52.3
30–34	40	18.7
35–39	12	5.6
≥40	4	1.9
**Education**		
Nil	136	63.6
Primary	54	22.4
Secondary	14	6.5
Tertiary	10	4.7
**Religion**		
Christian	200	93.5
Islam	2	0.9
Traditional	12	5.6
**Residence**		
Urban	51	23.8
Rural	163	76.2
**Occupation**		
Housewife	58	27.1
Farming	58	27.1
Petty trading	82	38.3
Artisan	11	5.1
Civil servant	5	2.3
**Marital status**		
Married	168	78.5
Unmarried	46	21.5
**Booking status**		
Booked	58	27.1
Unbooked	156	72.9
**Parity**		
0	92	43.0
1–2	76	35.5
3–4	24	11.2
≥5	22	10.3
**Total**	214	100

Majority of the women were primigravidae 92(43.0%); twenty-two (10.3%) were grand multiparous.

Feto/cephalopelvic disproportion was the commonest cause 106 (49.6%) of obstructed labour followed by cervical dystocia 45 (21.0%) as shown in Table 2. Caesarean section was the commonest (157, 73.4%) mode of delivery followed by laparotomy with a uterine repair only (24, 11.0%). A craniotomy was performed in three (1.5%) of the cases (not in the table).

**Table 2:** Maternal and neonatal outcome of obstructed labour

Outcome	Number	Percentage
**Maternal**		
Puerperal sepsis	48	22.4
Uterine rupture	46	21.5
PPH	13	6.1
Maternal death	12	5.6
Wound dehiscence	06	2.8
VVF	04	1.9
Foot drop	02	09
RVF	02	09
**Total**	**214**	**100**
**Neonatal**		
Normal Apgar score (≥7)	129	60.3
Birth asphyxia (≤4)	39	18.2
Neonatal sepsis	10	4.7
Fresh stillborn	18	8.4
Macerated baby	10	4.7
Early neonatal death	8	3.7
**Total**	**214**	**100**
**Neonatal weight (kg)**		
1.500–1.999	01	0.5
2.000–2.499	16	7.5
2.500–2.999	62	28.9
3.000–3.490	97	45.3
3.500–3.999	26	12.2
≥4.000	12	5.6
**Total**	**214**	**100**

VVF – Vesicovaginal fistula, RVF- rectovaginal fistula, PPH-post-partum haemorrhage

In Table 2, sixteen (7.5%) of the neonates weighed less than 2.5kg (all were retained second twin) while twelve (5.6%) weighed 4kg and above. Complications that were encountered include puerperal sepsis 48 (22.4%), ruptured uterus 46 (21.5%), vesicovaginal fistula in 4 (1.9%) and rectovaginal fistula in 2(0.9%) of the women (Table 2). There were twelve maternal deaths, during the study period, giving a maternal mortality ratio of 191 per 100,000 live births. The clinical causes of death were overwhelming sepsis and hypovolemic shock. All the cases of maternal death occurred among unbooked women that presented late at the hospital from remote villages, their labour was initially managed by a traditional birth attendant.

Table 2 showed that normal Apgar score was found in 129 (60.3%) of the babies while 39 (18.2%) had birth asphyxia. Thirty-six (36) of the neonates had perinatal death which gave a perinatal mortality rate of 168 per 1000 deliveries for the period under review.

As shown in Table 3, the booking status of the study population did not significantly influence the maternal outcome although being unbooked increased the odds of parturient developing puerperal sepsis, uterine rupture and PPH. Being unmarried, having no formal education, being unbooked, maternal age of 30 years or more and rural residence of parturient were strongly associated with increased odds of parturient developing post-partum complications although not significant. The odds of a neonate having abnormal Apgar score was significantly more with parturient being unbooked, residing in rural areas and 30 years or more (Table 4).

**Table 3:** Relationship between the socio-demographic/obstetrics characteristics of the study population and maternal outcome

Variable	Puerperal Sepsis		OR(95%CI)	Uterine rupture		OR(95%CI)	PPH		OR(95%CI)
	Yes	No		Yes	No		Yes	No	
**Booking**									
Unbooked	38	118	1.11(0.93–1.32)	44	110	1.46(1.28–1.65)	11	145	1.17(0.95–150)
Booked	10	48	Ref.	2	58	Ref.	2	56	Ref.
**Parity**									
0–2	28	140	Ref.	2	166	Ref.	4	154	Ref.
3–5	20	26	2.66(1.63–4.32)	44	2	80.34(20.23–319.05)	9	37	3.76((2.36–5.98)
**Age(years)**									
<30	36	122	Ref.	6	152	Ref.	4	154	Ref.
≥30	12	44	0.94(0.54–1.63)	40	16	9.13(5.63–14.74)	9	47	2.96(1.90–4.55)
**Education**									
Yes	6	72	Ref.	12	66	Ref.	5	73	Ref.
No	42	94	1.54(1.30–1.83)	34	102	1.21(0.98–1.50)	8	128	0.96(0.62–1.50)
**Marital** **status**							
Married	30	138	Ref.	36	132	Ref.	7	159	Ref.
Unmarried	18	28	2.22(1.35–3.65)	12	34	1.07(0.57–2.01)	6	42	2.20(1.15–4.21)
**Residence**									
Urban	8	43	Ref.	8	43	Ref.	3	49	Ref.
Rural	40	123	1.12(0.96–1.31)	38	125	1.11(0.94–1.30)	10	152	1.01(0.74–1.38)

**Table 4:** Relationship between the socio-demographic/obstetrics characteristics of the study population and neonatal outcome

Variables	Normal APGAR Score	Abnormal APGAR score	OR95%CI
**Booking**			
Unbooked	88	68	0.85 (0.72–0.99)*
Booked	41	17	Ref.
**Parity**			
0–2	91	77	Ref.
3–5	38	8	3.13(1.53–6.37)
**Age**			
<30	116	42	Ref.
≥30	13	43	0.19(0.11–0.34)*
**Education**			
Yes	40	38	Ref.
No	89	47	1.24(0.99–1.56)
**Marital status**			
Married	101	67	Ref.
Unmarried	28	18	1.02(0.60–1.73)
**Residence**			
Urban	43	8	Ref.
Rural	86	77	0.73(0.64–0.84)*

OR - odd ratio

* Significant

## Discussion

This current study is aimed at documenting the maternal and foetal outcome of cases of obstructed labour Ebonyi State University Teaching Hospital Abakaliki. Our study is able to show the great wastage associated with this preventable tragedy. The prevalence rate of obstructed labour in this study is 3.4% (95%CI 3.37–3.42) and it agrees with the rate of 3.2% reported by Aboyeji et al. in Ilorin^21^ but lower than the rate of 4.2% and 12.2% reported by Yusuf MA in Kano^22^ and Fantu et al. in Ethiopia18 respectively. Obstructed labour is a problem of developing economies and it is poverty-related. The low socio-economic status of the patient in this study gives credence to it and this is reinforced by the fact that a sizeable proportion of the patients could not afford the hospital fee for antenatal care.

The nulliparous patient was the most at risk of obstructed labour in this study, which is similar to the findings of Aboyeji et al. in Ilorin^21^ which shows that nulliparous patient was the most at risk with 43.0% of the cases recorded. Obstructed labour occurred in 6.6% of teenagers in our study. This finding is similar to the study by Melas et al in Gombe^23^ who recorded 6.8% of the patient who had obstructed labour to be teenagers. This may be because of early marriage in our society with its resultant high prevalence of teenage primigravida who goes into pregnancy and labour with the immature pelvis. The female pelvis attains full obstetric maturity at the age of 25–30 years 24.

In our study, 10.2% of grand multiparous patients had obstructed labour which is lower than the finding of Aboyeji et al. in Ilorin^21^. Obstructed labour can occur in a grand multiparous patient due to big babies as it is known that babies tend to get bigger with subsequent pregnancies 25. Spondylolisthesis can also be a contributing factor – a condition in which the fifth lumbar vertebrae rides forward on the upper surface of the first sacral vertebrae 26 thereby significantly reducing the anterior-posterior diameter of the pelvic inlet - as a result of exaggerated lumbar lordosis which the grand multiparas are prone to develop during pregnancy.

**Figure 1:**
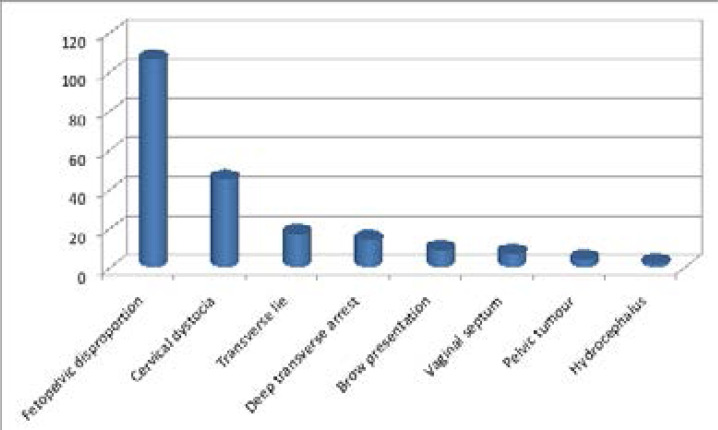
Causes of obstructed labour

Our study showed that fetopelvic disproportion was the commonest cause of obstructed labour accounting for 49.6% of the case studied. This had been the finding of Wall LL^27^ in Maiduguri with fetopelvic disproportion being te commonest cause of the obstructed labour. In his study, it accounted for 75.5% of the cases studied which is higher than 49.6% recorded in this study as shown in Figure 1. This could have been anticipated and prevented if the patient had booked for antenatal care or the disproportion detected when patients present in early labour before labour becomes obstructed^28^. Most of the patients studied were unbooked and did not benefit from the services of skilled birth attendance during pregnancy and labour. This could be a contributing/predisposing factor to their labour been obstructed. A skilled birth attendant will knowledgeably anticipate possible parturients that are likely to have obstructed labour during antenatal care evaluation and thus put measures in place in its prevention before or during labour.

Lower segment caesarean section was the commonest mode of delivery as 73.4% of the cases studied had the surgery. This is similar to the study by Yusuf MA in Kano^22^ who reported that lower segment caesarean section was the commonest mode of delivery as was recorded in 73.4% of the case studied. In the study of Aboyeji et al. in Ilorin^21^, lower segment caesarean section accounted for 86.6% of the mode of delivery which was similar to the result in this study.

Destructive surgery done was craniotomy in only 1.5% of the patient studied which agrees with the work of Yusuf et al^22^ in Kano and Aboyeji et al.^21^ in Ilorin which reported a craniotomy rate of 1.6% and 1.5% respectively. The perinatal mortality of 36 (16.8%) is lower than 52.9% recorded by Harrison KA29 in Zaria and 41.8% perinatal mortality reported by Aboyeji et al.^21^. This neonatal death might be the effect of prolonged labour and neonatal sepsis resulting from multiple unsterile vaginal examinations done before referral. The maternal mortality of 12 (5.6%) 95%CI 2.8–9.2% recorded in this study is compatible with earlier studies in Nigeria^8^, ^23^. This study has shown that obstructed labour is still a common obstetric problem in our environment and the situation is likely to be so for some time. This is because of the high prevalence of childhood infection and malnutrition in our society which affects pelvic bone development.

The most common maternal complications seen in our study include puerperal sepsis (22.4%), uterine rupture (21.5%) and PPH (6.1%). Other complications such as maternal death, wound dehiscence, vesicovaginal fistula (VVF), foot drop and rectovaginal fistula (RVF) accounted for 12.1% of the maternal complication. Some of these findings are similar to the work of Wonde et al. in Ethiopia where sepsis (25.3%), uterine rupture (16.5%) and anaemia from PPH (17.0%) accounted for the majority of maternal complications 30. Credence to our findings was also seen in the work of Anuma et al. 31 which reported on the contribution of puerperal sepsis and uterine rupture to maternal morbidity and mortality in the study area. The unbooked nature of most these women under review and the fact that the majority could not afford to procure their post-delivery antibiotics are some of the explainable reasons for the high burden of infectious morbidities in our review. Our study shows that parturient with no formal education has increased odds of puerperal sepsis than those with no formal education [OR = 1.11 95%CI 0.93–1.32]. Educated women are likely to be economically and socially empowered to break socio-cultural and financial barriers of poverty and will haveless risk of puerperal sepsis 32. These findings call on the need for prompt and astute care of these women and the use of broad-spectrum antibiotics to help reduce the development of infectious morbidities with its immediate and long term squeal.

As might be expected the majority of cases of uterine rupture occurred among parturient that are multiparous as they are at increased odds of uterine rupture following labour obstruction. Injudicious use of oxytocin and application of fundal pressure were some of the explainable reasons reported in these women. These women also travelled a long distance to access care in our facility thereby further increasing the risk of rupture. The risk of uterine rupture was 22% higher among the unbooked women compared to the booked women. It was also 22% higher among para 3–5 than para 0–2. This is similar to the study by Islam et al. which showed that uterine rupture was not common in the primigravida as primigravida's uterus meets labour obstruction with inertia whereas multigravid uterus meets obstruction with hypertonic uterine contraction 32. Multigravid uterus has more oxytocin receptors and are able to recruit more contracting units than primigravida uterus, thus hypertonic contraction in the face of obstruction 20, 32. During the process of labour, the uterus contracts and retracts with a buildup of pressure within the uterus. The aim is to expel the fetus through os vaginalis but with mechanical obstruction this does not occur. It will lead to the pathological stretching of the lower segment of the uterus, resulting in Bandl's ring formation and ultimately in uterine rupture if obstruction is not relieved 20. Apart from the hypertonic contraction, uterine rupture is more common in the multigravida's uterus because of increased number of fibrous tissue which are less amenable to stretch 20. Uterinerupture was also more on those with no formal education and those in the rural areas than in the urban area [OR 0.67 95%CI (0.34–1.43)]. Poor health-seeking behaviour and the far distance from the hospital are some of the factors that might be responsible for the above findings.

PPH remains a significant cause of maternal morbidity and mortality and has been estimated to occur in 3–5% of obstetrics population 33. Globally, PPH is responsible for about one-fourth of maternal deaths annually with the greater burden being borne in low-income countries 33, 34. The point prevalence of PPH in our study (PPH) was 6.1%. It is higher among the unbooked women than the booked women [/span>OR = 1.17 95% CI 0.91–1.50] and among para 3–5 than para 0–2 by 4.5% [OR = 3.76 95%CI 2.36–5.98]. This is as expected as these are risk factors earlier documented for PPH 35.

Maternal death accounts for 5.6% of the complications in this study. This was mainly due to puerperal sepsis, uterine rupture and PPH. This can be prevented through the provision of adequate and affordable antenatal care and skilled delivery care in the rural and semi-urban areas. Our rate of maternal death is higher than the 2% and 0.9% maternal death rate reported by Bako et al. (Maiduguri, Nigeria) 36 and Islam et al. in Bangladesh 32 respectively. The fact that our facility is the only tertiary hospital in the state and receiving the majority of the referrals could be an explainable reason for our finding. The study also shows that obstructed labour is one of the major causes of poor perinatal outcome. The perinatal outcome of 16.8% observed in this study is higher when compared to 7.18% reported in Maiduguri 36 but it is lower than other studies from India 37 where perinatal mortality of 22.68% was reported. The poor fetal outcome and perinatal deaths were as a result of prolonged labour and neonatal sepsis.

Obstructed labour with its attendant complications is better prevented than managed. Antenatal care by a skilled birth attendant offers a veritable opportunity of identification of women at risk thereby prompting a proactive action on its prevention. Majority of our study population were unbooked, never benefited from antenatal care and only presented in our facility for care because of inability to deliver at traditional birth attendant home. It is unfortunate as evidence has shown the importance of skilled birth attendance in Safe motherhood^13^. Training and retraining of traditional birth attendant, health education, campaign for antenatal care and early presentation in the hospital when in labour is important in reducing the burden of obstructed in Nigeria 38. Family and community attitude that inhibit the use of health facilities should be discouraged through intensive health education.

Provision of free or subsidized quality antenatal care and delivery facilities should be encouraged^39^ as our study has shown that some of these women attributed to non-attendance of antenatal care to lack of money. Family planning should be encouraged particularly for the grand multiparous patient to prevent unintended pregnancy. Prompt referral by the midwives to either a secondary or tertiary health facility of a parturient at risk of obstructed labour is important and education of these midwives on the use of partograph would help in early referral^17^. It is also important that the secondary and tertiary health care facilities should be adequately equipped and staffed to handle cases that are been referred to them. As a long-term measure, improvement in food security via availability, accessibility, utilization, and stability would go a long way in addressing the contribution of malnutrition to the burden of obstructed labour in Nigeria.

## Conclusion

This study has revealed the burden of obstructed labour and its complications in our environment. Majority of the study population were unbooked with cephalopelvic disproportion being the commonest cause. To alleviate these problems, the Ministry of Health and other responsible bodies need to make concerted efforts to increase antenatal care coverage. It would be good if antenatal care service is subsidized to make it affordable to the obstetric population in our country. We also recommend routine intrapartum assessment of cephalopelvic disproportion as an approach to preventing obstructed labour.

## Limitations of the study

One of the limitations of this study is that the maternal and neonatal outcome of women with obstructed labour was not compared with other women without obstructed labour. This would have helped to show if the outcomes recorded can be attributed solely to obstructed labour or other variables such as socio-cultural factors. Diagnosis of obstructed labour is influenced by its subjective nature and parturients with prolonged labour might have been diagnosed as a case of obstructed labour. However, an effort was made to limit the wrong diagnosis as the diagnosis of obstructed labour was made by either a consultant or a senior registrar. It is also limited by being a retrospective hospital-based study with its bias of documentation; the findings might not also be a true representation of the obstetrics population of the study area. Our hospital is a referral centre and our finding might be influenced by the concentration of difficult cases that were referred from the peripheral hospital.

## Declarations

### Availability of data and material

All data generated or analysed during this study are included in this published article.
